# 1,3,5-Trinitro-2,4-bis­(2-phenyl­ethen­yl)benzene

**DOI:** 10.1107/S1600536810034732

**Published:** 2010-09-04

**Authors:** Ze-Rong Guo, Hua-Bo Li, Fang Li

**Affiliations:** a State Key Laboratory of Explosion Science and Technology, Beijing Institute of Technology, Beijing 100081, People’s Republic of China

## Abstract

In the title compound, C_22_H_15_N_3_O_6_, the central benzene ring and one of the phenyl rings are essentially parallel to each other, making a dihedral angle of 1.35 (16)°. The dihedral angle between the two phenyl rings is 83.56 (19)°. Intra­molecular C—H⋯N and C—H⋯O hydrogen bonds occur. In the crystal, mol­ecules are linked through C—H⋯O hydrogen bonds. Furthermore, offset face-to-face π–π inter­actions with centroid–centroid distances of 3.644 (2) Å help to stabilize the crystal structure.

## Related literature

For the preparation, see: Peng *et al.* (1995 ▶). For general background to trinitro­benzene and its derivatives, see: Ott & Benziger (1987 ▶); Kuperman *et al.* (2006 ▶). The title compound may be useful as a high energy explosive, see: Peng *et al.* (1995 ▶). For a related structure, see: Bryden (1972 ▶).
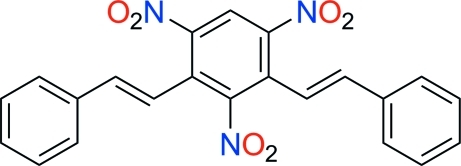


## Experimental

### 

#### Crystal data

C_22_H_15_N_3_O_6_
*M* *_r_* = 417.37
Triclinic, 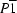

*a* = 7.0762 (14) Å
*b* = 8.6625 (17) Å
*c* = 16.717 (3) Å
α = 101.660 (3)°
β = 92.616 (3)°
γ = 105.122 (3)°
*V* = 963.5 (3) Å^3^
*Z* = 2
Mo *K*α radiation
μ = 0.11 mm^−1^
*T* = 293 K
0.32 × 0.28 × 0.22 mm

#### Data collection

Bruker SMART APEX CCD area-detector diffractometer
Absorption correction: multi-scan (*SADABS*; Bruker, 2003 ▶) *T*_min_ = 0.577, *T*_max_ = 1.000
5265 measured reflections
3363 independent reflections
2146 reflections with *I* > 2σ(*I*)
*R*_int_ = 0.020

#### Refinement

*R*[*F* ^2^ > 2σ(*F* ^2^)] = 0.082
*wR*(*F* ^2^) = 0.236
*S* = 0.98
3363 reflections
280 parameters
H-atom parameters constrained
Δρ_max_ = 0.66 e Å^−3^
Δρ_min_ = −0.26 e Å^−3^

### 

Data collection: *SMART* (Bruker, 2002 ▶); cell refinement: *SAINT-Plus* (Bruker, 2003 ▶); data reduction: *SAINT-Plus*; program(s) used to solve structure: *SHELXTL* (Sheldrick, 2008 ▶); program(s) used to refine structure: *SHELXTL*; molecular graphics: *ORTEP-3* (Farrugia, 1997 ▶); software used to prepare material for publication: *WinGX* (Farrugia, 1999 ▶).

## Supplementary Material

Crystal structure: contains datablocks I, global. DOI: 10.1107/S1600536810034732/wn2408sup1.cif

Structure factors: contains datablocks I. DOI: 10.1107/S1600536810034732/wn2408Isup2.hkl

Additional supplementary materials:  crystallographic information; 3D view; checkCIF report

## Figures and Tables

**Table 1:** Hydrogen-bond geometry (Å, °)

*D*—H⋯*A*	*D*—H	H⋯*A*	*D*⋯*A*	*D*—H⋯*A*
C16—H16⋯O2	0.93	2.60	3.398 (4)	144
C16—H16⋯N1	0.93	2.42	2.980 (4)	119
C18—H18⋯O5^i^	0.93	2.48	3.387 (4)	166

Symmetry code: (i) 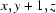.

**Fig. 1.**
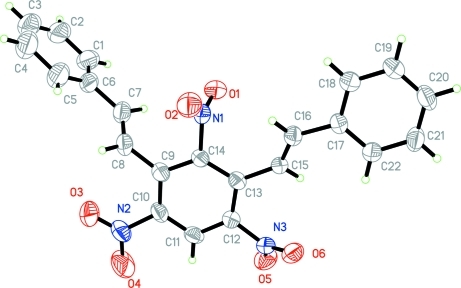
The molecular structure of the title compound with the atom numbering scheme. Displacement ellipsoids are drawn at the 50% probability level. H atoms are presented as a small spheres of arbitrary radius.

**Fig. 2.**
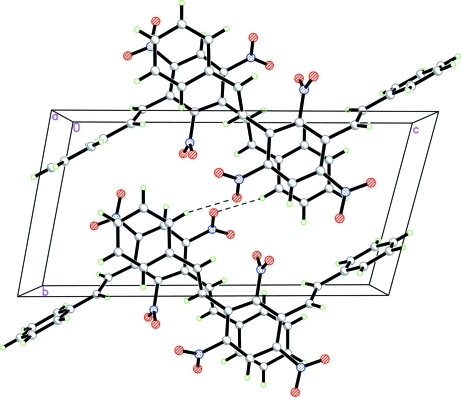
The packing of the title compound, viewed along the a-axis. Intermolecular hydrogen bonds are shown as dashed lines.

## supplementary crystallographic information

### Comment

Trinitrobenzene and its derivatives have been extensively reported for use as energetic materials (Ott & Benziger, 1987; Kuperman *et al.*, 2006). The title compound may be useful as a high energy explosive (Peng *et al.*, 1995), and here we present its crystal structure.

In the title compound (Fig. 1), the bond distances and bond angles are similar to those in 2,4,6-trintro-*m*-xylene (Bryden, 1972). The planes of two rings (C9—C14) and (C17—C22) are approximately parallel, with a dihedral angle of 1.35 (16)°. The two phenyl rings, (C1—C6) and (C17—C22), form a dihedral angle of 83.56 (19)°. The short distance of 3.644 (2) Å (symmetry code: -*x*,-*y*,1 - *z*) between the centroids of the two parallel rings (C9—C14) and (C17—C22) indicates the existence of offset face-to-face π-π interactions. Molecules are linked through C—H···O hydrogen bonds (Table 1), which help to stabilize the crystal structure. Intramolecular C—H···N and C—H···O hydrogen bonds are also present. There is a short intermolecular contact C15···C15 (1-*x*, -*y*, 1-*z*) of 3.185 (4) Å.

### Experimental

The title compound was synthesized using 2,4,6-trinitro-*m*-xylene and benzaldehyde as the starting materials, according to the literature method (Peng *et al.*, 1995). Single crystals suitable for *X*–ray diffraction were prepared by slow evaporation of a solution of the title compound in acetone at room temperature.

### Refinement

All H atoms were placed in calculated positions, with C—H = 0.93 Å; *U*_iso_(H) = 1.2*U*_eq_(C).

### Crystal data

**Table:**

C_22_H_15_N_3_O_6_	*Z* = 2
*M**_r_* = 417.37	*F*(000) = 432
Triclinic, *P*1	*D*_x_ = 1.439 Mg m^−^^3^
Hall symbol: -P 1	Mo *K*α radiation, λ = 0.71073 Å
*a* = 7.0762 (14) Å	Cell parameters from 1118 reflections
*b* = 8.6625 (17) Å	θ = 2.5–22.8°
*c* = 16.717 (3) Å	µ = 0.11 mm^−^^1^
α = 101.660 (3)°	*T* = 293 K
β = 92.616 (3)°	Block, colorless
γ = 105.122 (3)°	0.32 × 0.28 × 0.22 mm
*V* = 963.5 (3) Å^3^	

### Data collection

**Table:**

Bruker SMART APEX CCD area-detector diffractometer	3363 independent reflections
Radiation source: fine-focus sealed tube	2146 reflections with *I* > 2σ(*I*)
graphite	*R*_int_ = 0.020
phi and ω scans	θ_max_ = 25.0°, θ_min_ = 2.5°
Absorption correction: multi-scan (*SADABS*; Bruker, 2003)	*h* = −8→8
*T*_min_ = 0.577, *T*_max_ = 1.000	*k* = −10→9
5265 measured reflections	*l* = −18→19

### Refinement

**Table:**

Refinement on *F*^2^	Primary atom site location: structure-invariant direct methods
Least-squares matrix: full	Secondary atom site location: difference Fourier map
*R*[*F*^2^ > 2σ(*F*^2^)] = 0.082	Hydrogen site location: inferred from neighbouring sites
*wR*(*F*^2^) = 0.236	H-atom parameters constrained
*S* = 0.98	*w* = 1/[σ^2^(*F*_o_^2^) + (0.1716*P*)^2^] where *P* = (*F*_o_^2^ + 2*F*_c_^2^)/3
3363 reflections	(Δ/σ)_max_ < 0.001
280 parameters	Δρ_max_ = 0.66 e Å^−^^3^
0 restraints	Δρ_min_ = −0.26 e Å^−^^3^

### Special details

**Table:**

Geometry. All e.s.d.'s (except the e.s.d. in the dihedral angle between two l.s. planes) are estimated using the full covariance matrix. The cell e.s.d.'s are taken into account individually in the estimation of e.s.d.'s in distances, angles and torsion angles; correlations between e.s.d.'s in cell parameters are only used when they are defined by crystal symmetry. An approximate (isotropic) treatment of cell e.s.d.'s is used for estimating e.s.d.'s involving l.s. planes.
Refinement. Refinement of *F*^2^ against ALL reflections. The weighted *R*-factor *wR* and goodness of fit *S* are based on *F*^2^, conventional *R*-factors *R* are based on *F*, with *F* set to zero for negative *F*^2^. The threshold expression of *F*^2^ > σ(*F*^2^) is used only for calculating *R*-factors(gt) *etc*. and is not relevant to the choice of reflections for refinement. *R*-factors based on *F*^2^ are statistically about twice as large as those based on *F*, and *R*- factors based on ALL data will be even larger.

### Fractional atomic coordinates and isotropic or equivalent isotropic displacement parameters (Å^2^)

**Table:**

	*x*	*y*	*z*	*U*_iso_*/*U*_eq_	
O1	0.4557 (4)	0.2087 (3)	0.37673 (15)	0.0606 (7)	
O2	0.1502 (4)	0.1919 (3)	0.34477 (15)	0.0629 (7)	
O3	0.0777 (6)	−0.3754 (4)	0.12446 (17)	0.1042 (13)	
O4	0.0663 (6)	−0.5709 (4)	0.18422 (17)	0.0944 (11)	
O5	0.2954 (4)	−0.4565 (3)	0.47219 (15)	0.0599 (7)	
O6	0.1092 (4)	−0.3227 (3)	0.53578 (14)	0.0558 (7)	
N1	0.2829 (5)	0.1337 (3)	0.35892 (14)	0.0450 (7)	
N2	0.0966 (4)	−0.4259 (4)	0.18514 (18)	0.0576 (8)	
N3	0.2018 (4)	−0.3561 (3)	0.47819 (16)	0.0413 (6)	
C1	0.5959 (7)	0.1705 (4)	0.0976 (2)	0.0659 (11)	
H1	0.6973	0.1494	0.1272	0.079*	
C2	0.6401 (8)	0.2529 (5)	0.0357 (2)	0.0796 (13)	
H2	0.7691	0.2860	0.0230	0.096*	
C3	0.4936 (11)	0.2845 (6)	−0.0061 (3)	0.0892 (16)	
H3	0.5228	0.3398	−0.0482	0.107*	
C4	0.3043 (10)	0.2385 (6)	0.0110 (3)	0.0914 (16)	
H4	0.2057	0.2629	−0.0187	0.110*	
C5	0.2581 (7)	0.1525 (5)	0.0745 (2)	0.0739 (12)	
H5	0.1289	0.1199	0.0869	0.089*	
C6	0.4058 (6)	0.1181 (4)	0.11752 (19)	0.0533 (9)	
C7	0.3715 (6)	0.0306 (4)	0.18496 (19)	0.0502 (9)	
H7	0.4820	0.0413	0.2202	0.060*	
C8	0.2044 (5)	−0.0607 (4)	0.20150 (18)	0.0479 (8)	
H8	0.0901	−0.0756	0.1677	0.057*	
C9	0.1924 (4)	−0.1401 (4)	0.27196 (18)	0.0403 (7)	
C10	0.1482 (5)	−0.3101 (4)	0.26559 (18)	0.0417 (8)	
C11	0.1561 (4)	−0.3773 (4)	0.33316 (18)	0.0413 (8)	
H11	0.1338	−0.4899	0.3271	0.050*	
C12	0.1978 (4)	−0.2745 (3)	0.40967 (17)	0.0369 (7)	
C13	0.2335 (4)	−0.1039 (3)	0.42426 (17)	0.0352 (7)	
C14	0.2324 (4)	−0.0458 (3)	0.35209 (17)	0.0358 (7)	
C15	0.2759 (4)	−0.0028 (3)	0.50832 (17)	0.0351 (7)	
H15	0.3253	−0.0496	0.5470	0.042*	
C16	0.2545 (4)	0.1434 (4)	0.53706 (17)	0.0390 (7)	
H16	0.2098	0.1960	0.4999	0.047*	
C17	0.2962 (4)	0.2302 (3)	0.62389 (17)	0.0367 (7)	
C18	0.3403 (5)	0.3997 (4)	0.6440 (2)	0.0480 (8)	
H18	0.3405	0.4570	0.6026	0.058*	
C19	0.3838 (6)	0.4843 (4)	0.7246 (2)	0.0557 (9)	
H19	0.4147	0.5982	0.7372	0.067*	
C20	0.3820 (5)	0.4014 (4)	0.7870 (2)	0.0559 (9)	
H20	0.4124	0.4589	0.8414	0.067*	
C21	0.3350 (5)	0.2343 (4)	0.76815 (19)	0.0519 (9)	
H21	0.3313	0.1781	0.8102	0.062*	
C22	0.2930 (5)	0.1474 (4)	0.68758 (18)	0.0433 (8)	
H22	0.2624	0.0335	0.6757	0.052*	

### Atomic displacement parameters (Å^2^)

**Table:**

	*U*^11^	*U*^22^	*U*^33^	*U*^12^	*U*^13^	*U*^23^
O1	0.0682 (18)	0.0454 (14)	0.0558 (15)	−0.0073 (13)	−0.0013 (12)	0.0152 (11)
O2	0.0861 (19)	0.0507 (15)	0.0599 (16)	0.0323 (14)	0.0044 (13)	0.0132 (12)
O3	0.180 (4)	0.072 (2)	0.0320 (16)	−0.007 (2)	−0.0013 (17)	0.0030 (14)
O4	0.155 (3)	0.0493 (18)	0.0638 (19)	0.0272 (18)	−0.0128 (18)	−0.0140 (14)
O5	0.0746 (17)	0.0522 (15)	0.0633 (16)	0.0263 (13)	0.0098 (12)	0.0245 (12)
O6	0.0698 (16)	0.0471 (14)	0.0498 (15)	0.0096 (12)	0.0190 (12)	0.0151 (11)
N1	0.0638 (19)	0.0409 (15)	0.0280 (14)	0.0108 (15)	0.0040 (12)	0.0074 (11)
N2	0.068 (2)	0.0486 (19)	0.0429 (18)	0.0054 (15)	0.0006 (14)	−0.0056 (14)
N3	0.0465 (16)	0.0338 (14)	0.0398 (15)	0.0042 (12)	0.0049 (12)	0.0084 (11)
C1	0.096 (3)	0.047 (2)	0.053 (2)	0.015 (2)	0.020 (2)	0.0127 (17)
C2	0.125 (4)	0.057 (3)	0.051 (2)	0.011 (3)	0.022 (3)	0.015 (2)
C3	0.159 (5)	0.058 (3)	0.046 (2)	0.019 (3)	0.017 (3)	0.014 (2)
C4	0.147 (5)	0.075 (3)	0.057 (3)	0.041 (3)	−0.009 (3)	0.017 (2)
C5	0.110 (3)	0.065 (3)	0.053 (2)	0.030 (2)	0.006 (2)	0.020 (2)
C6	0.086 (3)	0.0400 (18)	0.0337 (18)	0.0197 (18)	0.0083 (17)	0.0050 (14)
C7	0.069 (2)	0.048 (2)	0.0352 (18)	0.0197 (18)	0.0029 (15)	0.0086 (14)
C8	0.059 (2)	0.051 (2)	0.0295 (17)	0.0140 (17)	−0.0004 (14)	0.0042 (14)
C9	0.0413 (17)	0.0392 (17)	0.0362 (17)	0.0080 (13)	0.0004 (13)	0.0038 (13)
C10	0.0440 (18)	0.0400 (17)	0.0330 (16)	0.0072 (14)	0.0008 (13)	−0.0035 (13)
C11	0.0452 (18)	0.0293 (16)	0.0442 (18)	0.0058 (13)	0.0052 (14)	0.0019 (13)
C12	0.0367 (16)	0.0353 (16)	0.0368 (17)	0.0074 (12)	0.0032 (12)	0.0074 (13)
C13	0.0317 (16)	0.0353 (16)	0.0356 (16)	0.0074 (12)	0.0013 (12)	0.0042 (12)
C14	0.0377 (17)	0.0320 (15)	0.0344 (16)	0.0067 (12)	0.0027 (12)	0.0039 (12)
C15	0.0393 (17)	0.0313 (16)	0.0318 (15)	0.0043 (12)	−0.0002 (12)	0.0083 (12)
C16	0.0436 (18)	0.0398 (17)	0.0335 (16)	0.0118 (13)	0.0001 (13)	0.0083 (13)
C17	0.0361 (16)	0.0396 (17)	0.0317 (16)	0.0097 (13)	0.0021 (12)	0.0031 (13)
C18	0.065 (2)	0.0358 (17)	0.0421 (18)	0.0130 (15)	0.0056 (15)	0.0073 (14)
C19	0.078 (2)	0.0366 (18)	0.046 (2)	0.0108 (17)	0.0036 (17)	0.0008 (15)
C20	0.072 (2)	0.050 (2)	0.0381 (19)	0.0164 (18)	−0.0009 (16)	−0.0058 (16)
C21	0.070 (2)	0.057 (2)	0.0328 (17)	0.0241 (18)	0.0047 (15)	0.0125 (15)
C22	0.055 (2)	0.0367 (16)	0.0390 (17)	0.0126 (14)	0.0058 (14)	0.0087 (13)

### Geometric parameters (Å, °)

**Table:**

O1—N1	1.217 (3)	C8—H8	0.9300
O2—N1	1.211 (3)	C9—C14	1.394 (4)
O3—N2	1.199 (4)	C9—C10	1.404 (4)
O4—N2	1.214 (4)	C10—C11	1.376 (4)
O5—N3	1.216 (3)	C11—C12	1.372 (4)
O6—N3	1.218 (3)	C11—H11	0.9300
N1—C14	1.481 (4)	C12—C13	1.401 (4)
N2—C10	1.472 (4)	C13—C14	1.398 (4)
N3—C12	1.465 (4)	C13—C15	1.469 (4)
C1—C2	1.376 (5)	C15—C16	1.311 (4)
C1—C6	1.382 (5)	C15—H15	0.9300
C1—H1	0.9300	C16—C17	1.471 (4)
C2—C3	1.341 (7)	C16—H16	0.9300
C2—H2	0.9300	C17—C18	1.385 (4)
C3—C4	1.356 (7)	C17—C22	1.397 (4)
C3—H3	0.9300	C18—C19	1.376 (4)
C4—C5	1.420 (6)	C18—H18	0.9300
C4—H4	0.9300	C19—C20	1.379 (5)
C5—C6	1.370 (5)	C19—H19	0.9300
C5—H5	0.9300	C20—C21	1.364 (5)
C6—C7	1.476 (5)	C20—H20	0.9300
C7—C8	1.315 (5)	C21—C22	1.381 (4)
C7—H7	0.9300	C21—H21	0.9300
C8—C9	1.475 (4)	C22—H22	0.9300
			
O2—N1—O1	126.0 (3)	C9—C10—N2	121.2 (3)
O2—N1—C14	117.4 (3)	C12—C11—C10	118.6 (3)
O1—N1—C14	116.5 (3)	C12—C11—H11	120.7
O3—N2—O4	122.9 (3)	C10—C11—H11	120.7
O3—N2—C10	119.5 (3)	C11—C12—C13	124.4 (3)
O4—N2—C10	117.5 (3)	C11—C12—N3	115.1 (3)
O5—N3—O6	124.5 (3)	C13—C12—N3	120.5 (2)
O5—N3—C12	116.7 (3)	C14—C13—C12	113.1 (3)
O6—N3—C12	118.8 (2)	C14—C13—C15	126.0 (3)
C2—C1—C6	122.2 (4)	C12—C13—C15	120.9 (3)
C2—C1—H1	118.9	C9—C14—C13	126.6 (3)
C6—C1—H1	118.9	C9—C14—N1	114.9 (3)
C3—C2—C1	118.7 (5)	C13—C14—N1	118.4 (2)
C3—C2—H2	120.7	C16—C15—C13	129.8 (3)
C1—C2—H2	120.7	C16—C15—H15	115.1
C2—C3—C4	122.1 (5)	C13—C15—H15	115.1
C2—C3—H3	118.9	C15—C16—C17	124.7 (3)
C4—C3—H3	118.9	C15—C16—H16	117.7
C3—C4—C5	119.3 (5)	C17—C16—H16	117.7
C3—C4—H4	120.3	C18—C17—C22	118.3 (3)
C5—C4—H4	120.3	C18—C17—C16	119.5 (3)
C6—C5—C4	119.4 (5)	C22—C17—C16	122.2 (3)
C6—C5—H5	120.3	C19—C18—C17	120.8 (3)
C4—C5—H5	120.3	C19—C18—H18	119.6
C5—C6—C1	118.3 (4)	C17—C18—H18	119.6
C5—C6—C7	123.0 (4)	C18—C19—C20	120.4 (3)
C1—C6—C7	118.6 (3)	C18—C19—H19	119.8
C8—C7—C6	128.2 (3)	C20—C19—H19	119.8
C8—C7—H7	115.9	C21—C20—C19	119.4 (3)
C6—C7—H7	115.9	C21—C20—H20	120.3
C7—C8—C9	122.1 (3)	C19—C20—H20	120.3
C7—C8—H8	118.9	C20—C21—C22	120.9 (3)
C9—C8—H8	118.9	C20—C21—H21	119.5
C14—C9—C10	114.8 (3)	C22—C21—H21	119.5
C14—C9—C8	120.5 (3)	C21—C22—C17	120.1 (3)
C10—C9—C8	124.7 (3)	C21—C22—H22	120.0
C11—C10—C9	122.3 (3)	C17—C22—H22	120.0
C11—C10—N2	116.5 (3)		
			
C6—C1—C2—C3	0.7 (6)	C11—C12—C13—C14	−3.0 (4)
C1—C2—C3—C4	0.2 (7)	N3—C12—C13—C14	177.4 (2)
C2—C3—C4—C5	−0.6 (7)	C11—C12—C13—C15	179.6 (3)
C3—C4—C5—C6	0.1 (6)	N3—C12—C13—C15	0.0 (4)
C4—C5—C6—C1	0.7 (5)	C10—C9—C14—C13	0.4 (5)
C4—C5—C6—C7	179.5 (4)	C8—C9—C14—C13	−177.6 (3)
C2—C1—C6—C5	−1.1 (5)	C10—C9—C14—N1	178.9 (3)
C2—C1—C6—C7	−180.0 (3)	C8—C9—C14—N1	0.8 (4)
C5—C6—C7—C8	18.2 (5)	C12—C13—C14—C9	2.8 (4)
C1—C6—C7—C8	−163.0 (4)	C15—C13—C14—C9	−179.9 (3)
C6—C7—C8—C9	−179.5 (3)	C12—C13—C14—N1	−175.6 (2)
C7—C8—C9—C14	65.4 (4)	C15—C13—C14—N1	1.7 (4)
C7—C8—C9—C10	−112.4 (4)	O2—N1—C14—C9	74.1 (3)
C14—C9—C10—C11	−3.9 (4)	O1—N1—C14—C9	−104.5 (3)
C8—C9—C10—C11	174.0 (3)	O2—N1—C14—C13	−107.4 (3)
C14—C9—C10—N2	178.0 (3)	O1—N1—C14—C13	74.1 (3)
C8—C9—C10—N2	−4.0 (5)	C14—C13—C15—C16	26.2 (5)
O3—N2—C10—C11	176.3 (3)	C12—C13—C15—C16	−156.8 (3)
O4—N2—C10—C11	−0.5 (5)	C13—C15—C16—C17	177.8 (3)
O3—N2—C10—C9	−5.6 (5)	C15—C16—C17—C18	156.0 (3)
O4—N2—C10—C9	177.7 (3)	C15—C16—C17—C22	−24.0 (5)
C9—C10—C11—C12	3.8 (5)	C22—C17—C18—C19	1.4 (5)
N2—C10—C11—C12	−178.1 (3)	C16—C17—C18—C19	−178.7 (3)
C10—C11—C12—C13	−0.1 (5)	C17—C18—C19—C20	−0.8 (5)
C10—C11—C12—N3	179.5 (3)	C18—C19—C20—C21	−0.5 (6)
O5—N3—C12—C11	47.8 (4)	C19—C20—C21—C22	1.2 (5)
O6—N3—C12—C11	−130.5 (3)	C20—C21—C22—C17	−0.6 (5)
O5—N3—C12—C13	−132.5 (3)	C18—C17—C22—C21	−0.7 (5)
O6—N3—C12—C13	49.1 (4)	C16—C17—C22—C21	179.4 (3)

### Hydrogen-bond geometry (Å, °)

**Table:**

*D*—H···*A*	*D*—H	H···*A*	*D*···*A*	*D*—H···*A*
C16—H16···O2	0.93	2.60	3.398 (4)	144
C16—H16···N1	0.93	2.42	2.980 (4)	119
C18—H18···O5^i^	0.93	2.48	3.387 (4)	166

Symmetry codes: (i) *x*, *y*+1, *z*.
